# Supplementary material for: Knowledge, attitudes, and barriers to HIV testing among youth in Kumba, Cameroon: A cross-sectional qualitative community-based focus group study
Source: PLoS One. 2025 Nov 25;20(11):e0337099. doi: 10.1371/journal.pone.0337099 (PMC12646420; doi:10.1371/journal.pone.0337099)
Supplement: S1 Appendix — (PDF) [file pone.0337099.s001.pdf]

S1 Appendix: FGD Checklist (DOC) Data Instrument/Focus Group Discussion (FGD) Checklist

Topic: Knowledge, Attitudes, and Barriers to HIV Testing Among Youth in Kumba, Cameroon: A Cross-Sectional Qualitative Community-Based Focus Group Study

Location: ……………………………..………..

Date: ………..………………

Group Name: ………………………………………

Starting time: ………………

Was everyone able to participate equally? ☐ Yes, ☐ No

1. Introduction

Clarification of Objectives

- Briefly explain the purpose of the FGD (that is, to explore the knowledge, attitudes, and practices related to HIV testing among youths in Kumba).
- Explain confidentiality and ensure participants' anonymity.
- Establish ground rules (for instance, respect all opinions, no interrupting, one person speaks at a time, people speak freely but remain respectful).

Participant Consent

- Confirm participants' voluntary participation
- Obtain verbal consent before proceeding.

2. Demographic Information

- Age range (in years): 18-35 years (☐ < 20, ☐ 20 – 29, ☐ ≥ 30)
- Sex: Male/Female (# Males: ………… # Females: …………)
- Educational background: No Formal Education, Primary, Secondary, Tertiary (All strata)
- Employment status: No exception
- Marital status: Single/Married
- Religion: No exception

3. Knowledge of HIV and HIV Testing

Understanding HIV:

- What do you know about HIV? (Symptoms, transmission, prevention)
- How do you think HIV is transmitted? (Probe for common misconceptions)
- What is the difference between HIV and AIDS?

HIV Testing Awareness:

- Are you aware of the HIV testing services available here in Kumba?
- Can you describe what happens during an HIV test? (Probe for clarity and correct understanding)
- What are the benefits of getting tested for HIV?
- Have you ever been tested for HIV?

Knowledge Sources:

- Where did you get your information about HIV and HIV testing? (Family, friends, media, health professionals, others)
- What sources do you trust the most for HIV-related information?

4. Attitudes Toward HIV Testing

Personal Views on HIV Testing:

- How do you feel about getting tested for HIV?
- Would you encourage someone to get tested for HIV? Why or why not?

Social and Cultural Attitudes:

- What do you think the community's general attitude is toward HIV testing?
- Are there any stigmas or taboos related to HIV testing in your community? Please explain.

Fear and Misconceptions:

- What are the reasons someone might avoid getting tested for HIV in your community?
- Do you think people are afraid of the result? If yes, why?

5. Practices Regarding HIV Testing

Personal Experience with HIV Testing:

- Have you ever been tested for HIV? If yes, what was your experience like?
- If you haven't been tested, what would make you decide to get tested?
- What factors influence your decision to get tested for HIV or not?

Frequency of Testing:

- How often do you think people in your age group should get tested for HIV?

Barriers to Testing:

- Are there any barriers (for instance, cost, lack of privacy, fear of stigma, accessibility) that prevent people from getting tested in your community?

Support Systems:

- Are there any support systems (friends, family, and health workers) that can encourage youth to get tested?

- Who do you think would be the best person to support someone getting tested for HIV?

6. Community-Based HIV Testing Initiatives

Awareness of Local Programmes:

- Do you know of any HIV testing campaigns or programmes in Kumba?
- Have you participated in any such programmes or seen others do so?

Effectiveness of Awareness Campaigns:

- How effective do you think community-based HIV testing campaigns are in encouraging testing among youth? Why or why not?

Suggestions for Improvement:

- How can HIV testing services be improved here in Kumba?
- What methods or strategies do you think would encourage more youths to get tested for HIV?

7. Closing Remarks

Summary of Key Insights:

- Recap the main points discussed.

Final Questions:

- Do you have any other thoughts or ideas on HIV testing that you would like to share?

Gratitude and Conclusion:

- Thank participants for their time and valuable insights.

Ending time of the FGD: ………………………………..
