# Supplementary material for: Knowledge, attitudes, and barriers to HIV testing among youth in Kumba, Cameroon: A cross-sectional qualitative community-based focus group study
Source: PLoS One. 2025 Nov 25;20(11):e0337099. doi: 10.1371/journal.pone.0337099 (PMC12646420; doi:10.1371/journal.pone.0337099)
Supplement: S3 Appendix — (PDF) [file pone.0337099.s003.pdf]

S3 Appendix. COREQ Checklist

Title: Knowledge, Attitudes, and Barriers to HIV Testing Among Youth in Kumba, Cameroon: A Cross-Sectional Qualitative Community-Based Focus Group Study

Section/Topic	Item No	Checklist item	Response for this study	Reported in Section
		**Domain 1: Research team and reflexivity**		
		Personal Characteristics		
Interviewer/facilitator	1	Which author/s conducted the interview or focus group? Interviewer/facilitator	Marie Clarie Fien Ndim and Diane Zinkeng Tongwa conducted the focus group discussions (FGDs) with support from a field co-facilitator (HIV Clinical Supervisor, not an author).	Methods
Credentials	2	What were the researcher's credentials? E.g. PhD, MD	Frederick Nchang Cho (MSc, BMLS, BSc);<br>Marie Clarie Fien Ndim (HND);<br>Diane Zinkeng Tongwa (HND);<br>Christabel Afor Tatah (BSN);<br>Franklin Ngwesse Ngome (BSN);<br>Eugine Mbuh Nyanjoh (MSc, BMLS);<br>Andrew N Tassang (MD, PhD).	Methods
Occupation	3	What was their occupation at the time of the study?	Frederick Cho – Academic/Field Supervisor, Data Analyst;<br>Marie Ndim & Diane Tongwa – Field Researchers/Facilitators;<br>Christabel Tatah & Franklin Ngome – Academic Supervisors;	Methods

			Eugine Nyanjoh & Andrew Tassang – General Supervisors.	
Gender	4	Was the researcher male or female?	Frederick Cho – Male;<br>Marie Ndim – Female;<br>Diane Tongwa – Female;<br>Christabel Tatah – Female;<br>Franklin Ngome – Male;<br>Eugine Nyanjoh – Male;<br>Andrew Tassang – Male.	Methods
Experience and training	5	What experience or training did the researcher have? Relationship with participants	All field researchers had prior training in qualitative data collection, HIV prevention, and youth health engagement. The supervisors had experience in clinical research, epidemiology, and qualitative analysis.	Methods
		**Relationship with participants**		
Relationship established	6	Was a relationship established prior to study commencement?	No personal relationship existed prior to recruitment. Participants were approached during mobilisation within the community.	Methods
Participant knowledge of the interviewer	7	What did the participants know about the researcher? e.g. personal goals, reasons for doing the research	Participants were informed that the research team were public health professionals conducting a study to understand youth perspectives on HIV testing to improve access and awareness.	Methods
Interviewer characteristics	8	What characteristics were reported about the interviewer/facilitator? e.g. Bias, assumptions, reasons and interests in the research topic	Facilitators were trained public health professionals, fluent in English and Pidgin, neutral in facilitation, and	Methods

			interested in improving youth access to HIV testing services.	
		**Domain 2: study design**		
		Theoretical framework		
Methodological orientation and Theory	9	What methodological orientation was stated to underpin the study? e.g. grounded theory, discourse analysis, ethnography, phenomenology, content analysis	Qualitative descriptive study; thematic analysis using Braun & Clarke framework.	Methods
		Participant selection		
Sampling	10	How were participants selected? e.g. purposive, convenience, consecutive, snowball	Purposive sampling across gender, age, education, and occupation.	Methods
Method of approach	11	How were participants approached? e.g. face-to-face, telephone, mail, email	Face-to-face through community leaders and youth associations.	Methods
Sample size	12	How many participants were in the study?	75 participants across nine FGDs.	Methods / Results
Non-participation	13	How many people refused to participate or dropped out? Reasons?	Three declined due to scheduling conflicts; no dropouts during FGDs.	Methods
Setting of data collection	14	Where was the data collected? e.g. home, clinic, workplace	Community halls and youth meeting centres in Kumba II municipality.	Methods
Presence of non-participants	15	Was anyone else present besides the participants and researchers?	HIV Clinical Supervisor (not an author) co-facilitated some FGDs.	Methods
Description of sample	16	What are the important characteristics of the sample? e.g. demographic data, date	Youth aged 18–35 years, diverse in education and occupation. Demographics recorded.	Results
		Data collection		
Interview guide	17	Were questions, prompts, guides provided by the authors? Was it pilot tested?	Semi-structured FGD guide developed by authors, reviewed by experts, and	Methods

			pilot-tested with 5 youths outside study area.	
Repeat interviews	18	Were repeat interviews carried out? If yes, how many?	No	Methods
Audio/visual recording	19	Did the research use audio or visual recording to collect the data?	Audio recording was planned but not conducted due to logistical constraints	Methods
Field notes	20	Were field notes made during and/or after the interview or focus group?	Yes, detailed field notes were taken during and immediately after each session.	Methods
Duration	21	What was the duration of the interviews or focus group?	60–90 minutes per FGD.	Methods
Data saturation	22	Was data saturation discussed?	Yes, reached after ninth FGD.	Methods
Transcripts returned	23	Were transcripts returned to participants for comment and/or correction?	No transcript return, but summary findings were discussed with community representatives for validation.	Methods
		**Domain 3: analysis and findings**<br>**Data analysis**		
Number of data coders	24	How many data coders coded the data?	Two coders: Frederick Nchang Cho and Marie Clarie Fien Ndim.	Methods
Description of the coding tree	25	Did authors provide a description of the coding tree?	Yes, primary and secondary themes were described, following Braun & Clarke’s thematic analysis framework.	Methods
Derivation of themes	26	Were themes identified in advance or derived from the data?	Themes were derived inductively from the data.	Methods
Software	27	What software, if applicable, was used to manage the data?	NVivo Version 14.	Methods

Participant checking	28	Did participants provide feedback on the findings?	No direct participant feedback; summary findings shared with community leaders for context.	Methods / Results
		Reporting		
Quotations presented	29	Were participant quotations presented to illustrate the themes / findings? Was each quotation identified? e.g. participant number	Yes, verbatim quotations presented with participant identifiers (FGD number and gender, e.g., FGD1-F, FGD3-M).	Results
Data and findings consistent	30	Was there consistency between the data presented and the findings?	Yes, data were consistent with findings, supported by participant quotations.	Results
Clarity of major themes	31	Were major themes clearly presented in the findings?	Yes, major themes such as knowledge gaps, stigma, confidentiality, attitudes, and barriers were clearly described.	Results
Clarity of minor themes	32	Is there a description of diverse cases or discussion of minor themes?	Yes, minor themes including gender-specific perceptions and social norms were discussed.	Results / Discussion
