# Supplementary material for: Knowledge, attitudes, and barriers to HIV testing among youth in Kumba, Cameroon: A cross-sectional qualitative community-based focus group study
Source: PLoS One. 2025 Nov 25;20(11):e0337099. doi: 10.1371/journal.pone.0337099 (PMC12646420; doi:10.1371/journal.pone.0337099)
Supplement: Attachment — Submitted filename: Response to Reviewers.pdf [file pone.0337099.s007.pdf]

Thursday, 23 October 2025

Rebuttal Letter to Editors and Peer Reviewers

Manuscript Title: Knowledge, Attitudes, and Barriers to HIV Testing Among Youth in Kumba, Cameroon: A Cross-Sectional Qualitative Community-Based Focus Group Study

Journal: *PLOS ONE*

Submission ID: PONE-D-25-23676

Corresponding Authors: Frederick Nchang Cho and Marie Clarie Fien Ndim

Dear Sir/Madam,

We are grateful for the opportunity to revise our manuscript and thank you sincerely for the thoughtful, detailed, and constructive feedback provided. Your insights have significantly helped us to enhance the clarity, scientific rigour, and relevance of our work.

In this letter, we present a detailed, point-by-point response to all comments raised by the academic editor and reviewer. All changes have been clearly indicated in the revised manuscript, with line numbers referring to the clean version**,** unless otherwise stated.

Summary of Submission Items (as per editorial instructions):

- This Response to Reviewers letter (submitted as a separate file).
- Revised Manuscript with Track Changes (labelled accordingly).
- Clean Manuscript without Track Changes (labelled "Manuscript").

General Revisions

In addition to addressing all reviewer comments, we have made the following general updates:

- Corrected the ORCID ID for Author One.
- Added the ORCID ID for Author Four.
- Adjusted the affiliations of Authors One and Seven.
- Corrected the name of Author Six.
- Spell-checked the manuscript and corrected grammatical errors.
- Adjusted the manuscript title to improve clarity and alignment with journal standards.

We have also included the anonymised data set (S2 Appendix. FGD Dataset Minimal Anonymised (XLSX)), as well as the COREQ (Consolidated Criteria for Reporting Qualitative Research) checklist for the focus group discussions as supplementary material (S3 Appendix. COREQ Checklist (PDF)). For consistency, we also included the "Definition of Key Terms", just before study population.

We believe these revisions and clarifications have significantly strengthened the manuscript. We are confident that the updated version provides a more accurate and comprehensive representation of our findings.

Please find our detailed, point-by-point responses to each reviewer's comments attached below, with specific line numbers and descriptions of the corresponding revisions.

We sincerely appreciate the reviewers' detailed and thoughtful suggestions. We believe the revisions have significantly improved the manuscript's clarity, depth, and value. Thank you for your consideration.

Sincerely,

Frederick Nchang Cho[1, 2] (nchang.cho@gmail.com) and

Marie Clarie Fien Ndim[1] (Marieclairendim@gmail.com), on behalf of all co-authors

[1]RAPHA Higher Institute of Health and Biomedical Sciences, Kumba - Cameroon.

[2]Cameroon Baptist Convention Health Services - HIV free/Strengthening Public Health Laboratory Systems, Douala, Cameroon.

Academic Editor: Point-by-Point Response

S/N	Reviewer's comment(s)	Authors' response(s)	Line(s)
	Methods		
1.	Correct the study dates to reflect the actual period of data collection,	The study dates have been corrected.	132
2.	Clarify the sampling strategy: if purposive sampling was used, remove references to random sampling,	Sampling clarified as multistage with purposive selection at final stage.	172 – 174
3.	Explicitly discuss data saturation and how it was determined.	Explanation of data saturation process added.	195 – 200
	Results		
4.	Include verbatim participant quotes to illustrate each theme.	Participant quotes added for each major theme.	275 – 278, 286 – 288, 295 – 296, 310 – 311, 320 – 323, 336 – 337, 367 – 369, & 377 – 380
5.	Clarify whether percentages represent coded response frequencies rather than statistical generalizations.	Clarified that percentages represent coded response frequencies.	250 – 252
6.	Reformat Figure 2 and correct decimal errors.	Figures 2 and 3 reformatted; decimal issues and footers corrected.	361 – 363, 384 – 386
	References		
7.	Replace Wikipedia and other non-peer-reviewed sources with official or peer-reviewed references. Verify all references exist and are accessible; remove or update any future-dated citations.	All sources reviewed. No non-peer-reviewed or future-dated references remain.	--
	Data availability		
8.	Provide a Data Availability Statement that complies with PLOS ONE policy. Anonymised transcripts, coding framework, or minimal dataset should be deposited in a public repository (e.g., Dryad, Figshare). If full transcripts cannot be shared, provide a clear justification.	Data statement revised to comply. Full transcripts not shared due to ethical restrictions. Anonymised dataset and coding framework submitted as S1 Dataset (Excel). Full data available upon request with ethics approval.	525 – 532
	Ethics		
9.	Add detail on how confidentiality was maintained during FGDs (e.g., anonymization, secure storage).	Explained confidentiality procedures, including anonymisation, secure storage, and NDAs with facilitators.	331 – 332
10.	Your ethics statement should only appear in the Methods section of your manuscript. If your ethics statement is written in any section besides the Methods, please move it to the Methods section and delete it from any other section. Please ensure that your ethics statement is included in your manuscript, as the ethics statement entered into the	Ethics statement moved to Methods section only.	320 – 332

	online submission form will not be published alongside your manuscript.		
	Presentation		
11.	Proofread for grammar, tense consistency, and formatting, Ensure all tables/figures are properly titled, numbered, and referenced, Upload the FGD guide/checklist as supplementary material.	Manuscript fully proofread. Tables and figures properly labelled.	--

Reviewer 1: Point-by-Point Response

S/N	Reviewer's comment(s)	Authors' response(s)	Line(s)
	Title		
1.	I recommend including the study design in the manuscript title to improve clarity and transparency. For example, the title could be revised as: *Knowledge, Attitudes and Barriers to HIV Testing Among Youth in Kumba: A Cross-Sectional Qualitative Community-Based Study.*	Title updated to: *Knowledge, Attitudes, and Barriers to HIV Testing Among Youth in Kumba, Cameroon: A Cross-Sectional Qualitative Community-Based Focus Group Study.*	1-3
	Abstract		
2.	Please include "Aim" as a subheading in the abstract to clearly present the study's objective and improve readability.	Added "Objective" subheading, consistent with *PLOS ONE* format.	41
3.	Please report the number of male and female participants to provide a clearer demographic overview.	Updated to: 75 youth (52 females, 23 males).	44
	Introduction		
4.	Please spell out the term CD4 in full (for example, CD4 T-lymphocyte count) when it first appears, before using the abbreviation.	Defined as "CD4 T-lymphocyte cells."	68 – 69
5.	Please add an appropriate in-text reference to support the statement and strengthen the evidence base.	WHO (2022) and UNAIDS (2023) references added.	72
6.	Add a subheading titled "Evidence Worldwide" to improve organization and readability.	Not added to maintain alignment with PLOS ONE formatting guidelines.	--
7.	Include an appropriate in-text reference to support the statement 'Over 80% of adolescents living with HIV reside in this region…'	Relevant references already included.	86
8.	Define the term youth clearly, including the specific age range used in the study, to enhance clarity and consistency.	Defined as individuals aged 15–35 years (see section "Definition of Key Terms").	149
9.	Add a relevant in-text reference to support the statement.	References [18, 19] added.	96 – 97
10.	Add an appropriate reference to strengthen the manuscript's credibility.	Reference [24] added.	110
11.	Please check whether the problems identified in Kumba have been sufficiently studied or if they truly represent a gap in the literature. Also clarify whether references 25 and 26 specifically highlight research gaps in Kumba itself or in neighbouring countries.	Literature reviewed. Research gap in Kumba confirmed.	112
	Methods		
12.	Consider revising the Study Area section to remove or condense information that is not directly relevant, focusing on details essential to understanding the study context.	Non-essential details removed; retained youth-related context.	135 – 138
13.	Please add an appropriate in-text reference to support the statement.	NIS (National Institute of Statistics) reference added.	143
14.	Kindly justify the choice of the multistage sampling strategy and provide a clear definition for clarity.	Sampling strategy clarified with four-stage explanation and justification.	169 – 172
15.	Please define what is meant by "active youth groups.	Now defined in "Definition of Concepts."	151 – 152

16.	Specify whether data were collected by the researcher, HIV clinic staff, or psychosocial staff.	Lead researcher and trained assistants conducted FGDs under HIV clinic supervision.	205 – 210
17.	Report the Cronbach's alpha value for the redesigned questions to indicate the instrument's reliability.	Not applicable. Semi-structured guide used for qualitative FGDs. Pretested for clarity.	
18.	Clarify whether the facilitator and note-taker were HIV experts, describe the meeting location, and reference the figure appropriately. Provide the full term of the abbreviation **FGD** (Focus Group Discussion) in the footnote of **Figure 1** for clarity.	Facilitators trained in qualitative research; FGDs held in private community halls. Figure 1 updated to define FGD.	314
	Results		
19.	Please specify who was included from the six neighborhoods.	Participants were youth aged 18–35 years from six neighbourhoods. Data collected from four due to contextual challenges. Representativeness maintained.	251 - 257
20.	Provide the full term of **FGD** (Focus Group Discussion) in the footnote of **Table 1**.	Footnote now fully defines the abbreviation as requested.	260
21.	Regarding **Tables 1–5**, please clarify whether statistical comparisons were performed between groups. If the tables present only frequencies and percentages as descriptive statistics, p-values may not be necessary. However, if group comparisons were conducted, include the corresponding p-values in the tables.	Only descriptive frequencies reported; no regression or p-values appropriate for qualitative data.	--
22.	The titles of **Tables 3 and 4** should be more self-explanatory. Please revise them to clearly reflect the content and variables presented.	Table 3 → "Attitudes and Perceptions of HIV Testing Among Youth Participants."<br><br>Table 4 → "Self-Reported Practices and Motivations for HIV Testing Among Youth Participants."	422, 456
23.	Consider using regression modeling to assess associations between variables. Implementing an appropriate regression model could provide stronger evidence and control for potential confounding factors.	Not applicable to qualitative FGD data; analysis is descriptive and thematic.	
	Limitations		
24.	As part of the study's limitations, please consider discussing the timeline of the study, the cross-sectional design, and the background or training of the data collectors, as these factors may influence interpretation and generalizability of the findings.	Limitations revised to include these aspects.	598 – 602
