# Supplementary material for: Knowledge, attitudes, and barriers to HIV testing among youth in Kumba, Cameroon: A cross-sectional qualitative community-based focus group study
Source: PLoS One. 2025 Nov 25;20(11):e0337099. doi: 10.1371/journal.pone.0337099 (PMC12646420; doi:10.1371/journal.pone.0337099)
Supplement: Attachment — Submitted filename: Final review HIV in Kumba 8 OCT 25PONE-D-25-23676 Zainab.docx [file pone.0337099.s004.docx]

**To:** The Editor
**PLOS ONE Journal**
**Date:** 10 October 2025

**Subject:** Reviewer Report – Research Article
**Title:** *Knowledge, Attitudes and Barriers to HIV Testing Among Youth in Kumba: A Community-Based Focus Group Study*
**Manuscript ID:** PONE-D-25-23676

**Dear Editor,**

I would like to acknowledge and sincerely appreciate the efforts of Mr. Frederick Nchang Cho, the corresponding and submitting author, and the entire research team for conducting this important study. Their work addresses a critical issue in understanding the knowledge, attitudes, and barriers to HIV testing among youth in Kumba, Cameroon, through a community-based approach. The study demonstrates thoughtful design and a strong commitment to improving practice and the quality of care.

I am pleased to provide my reviewer report for the above-mentioned manuscript. This study focuses on a highly relevant public health topic with potential to contribute valuable insights into HIV prevention and youth health promotion.

Please find below my detailed comments for the authors’ consideration.

**Reviewer Comments**

**Title**
I recommend including the study design in the manuscript title to improve clarity and transparency. For example, the title could be revised as:
*Knowledge, Attitudes and Barriers to HIV Testing Among Youth in Kumba: A Cross-Sectional Qualitative Community-Based Study.*

**Abstract**
**Line 36:** Please include “Aim” as a subheading in the abstract to clearly present the study’s objective and improve readability.

**Results**
**Line 44:** Please report the number of male and female participants to provide a clearer demographic overview.

**Introduction**
**Line 64:** Please spell out the term CD4 in full (for example, CD4 T-lymphocyte count) when it first appears, before using the abbreviation.
**Line 71:** Please add an appropriate in-text reference to support the statement and strengthen the evidence base.
**Line 81:** Add a subheading titled “Evidence Worldwide” to improve organization and readability.
**Line 82:** Include an appropriate in-text reference to support the statement.
**Line 87:** Define the term youth clearly, including the specific age range used in the study, to enhance clarity and consistency.
**Line 95:** Add a relevant in-text reference to support the statement.
**Line 108:** Add an appropriate reference to strengthen the manuscript’s credibility.
**Lines 108–110:** Please check whether the problems identified in Kumba have been sufficiently studied or if they truly represent a gap in the literature. Also clarify whether references 20 and 21 specifically highlight research gaps in Kumba itself or in neighboring countries.

**Study Area**
**Lines 137–141:** Consider revising the Study Area section to remove or condense information that is not directly relevant, focusing on details essential to understanding the study context.
**Line 145:** Please add an appropriate in-text reference to support the statement.

**Sampling Method**
**Line 164:** Kindly justify the choice of the multistage sampling strategy and provide a clear definition for clarity.
**Line 180:** Please define what is meant by “active youth groups.”

**Data Instruments and Data Collection**
**Line 190:** Specify whether data were collected by the researcher, HIV clinic staff, or psychosocial staff.
**Line 193:** Report the Cronbach’s alpha value for the redesigned questions to indicate the instrument’s reliability.
**Figure 1:** Clarify whether the facilitator and note-taker were HIV experts, describe the meeting location, and reference the figure appropriately.
Provide the full term of the abbreviation **FGD** (Focus Group Discussion) in the footnote of **Figure 1** for clarity.

**Sociodemographic Characteristics of FGD Participants**
**Line 220:** Please specify who was included from the six neighborhoods.
Provide the full term of **FGD** (Focus Group Discussion) in the footnote of **Table 1**.
Regarding **Tables 1–5**, please clarify whether statistical comparisons were performed between groups. If the tables present only frequencies and percentages as descriptive statistics, p-values may not be necessary. However, if group comparisons were conducted, include the corresponding p-values in the tables.
The titles of **Tables 3 and 4** should be more self-explanatory. Please revise them to clearly reflect the content and variables presented.
Consider using regression modeling to assess associations between variables. Implementing an appropriate regression model could provide stronger evidence and control for potential confounding factors.

**Limitations**
**Line 409:** As part of the study’s limitations, please consider discussing the timeline of the study, the cross-sectional design, and the background or training of the data collectors, as these factors may influence interpretation and generalizability of the findings.

**Conclusion**
I appreciate the authors’ efforts in addressing this important public health issue. The above comments aim to enhance the manuscript’s clarity, methodological rigor, and overall presentation.

Finally, I would like to thank the journal and its editorial team for giving me the opportunity to review this valuable work.

**Kind regards**
Zainab Mahmood Al-Zadjali
Sultan Qaboos University
Muscat, Oman
Email: [zainab.alzadjali@yahoo.com](mailto:zainab.alzadjali@yahoo.com)
